# Rapid diagnosis of different cardiovascular disease events from early released cardiac biomarkers, cTnI, BNP, and CRP, by biosensor technology

**DOI:** 10.3389/fcvm.2025.1600695

**Published:** 2025-12-17

**Authors:** Razi Ullah, Mubassir Khan, Yin Huang, Guixue Wang

**Affiliations:** 1 Key Laboratory for Biorheological Science and Technology of Ministry of Education, National Local Joint Engineering Lab for Vascular Implants, College of Bioengineering, Chongqing University, Chongqing, China; 2 Institute of Panvascular Biology, Jin Feng Laboratory, Chongqing, China; 3 Department of Gastrointestinal Surgery, The First Affiliated Hospital of Chongqing Medical University, Chongqing, China

**Keywords:** point-of-care, cardiovascular diseases, cardiac failure, cardiac troponins I (cTnI), B type natriuretic peptide (BNP), C-reactive protein (CRP)

## Abstract

Cardiovascular disease (CVD) represents a significant global health challenge, making the detection of cardiac biomarkers crucial for early diagnosis and tailored treatment strategies. This research aims to transpire a point-of-care (POC) test using a biosensor for CVDs that will be fast and pragmatic for immediate use in acute and resource-constrained environments. Traditional techniques such as enzyme-linked immunosorbent assay and polymerase chain reaction, although very precise, are time-consuming, labor intensive, and not suitable for use in urgent care; however, optical nano biosensors provide rapid, highly selective, and sensitive detection capabilities. The optical nano biosensors produce biological signals that convey light signals as analytes interact with bioreceptors. Optical nano biosensors offer various benefits, including effortless monitoring, inexpensiveness, a broad detection spectrum, and excellent sensitivity with no interference. An optical nano biosensor platform represents an effective method for point-of-care detection of cardiac biomarkers, characterized by a low detection limit. To propose a realistic reference, this study assesses a prompt POC test, which identifies important cardiac biomarkers, such as cardiac troponins (cTnI), B-type natriuretic peptide (BNP), and C-reactive protein (CRP), which together provide an all-encompassing confinement of myocardial injury, cardiac stress, and inflammation. Subsequently, the test was performed using a random patient population; the accuracy of the test was established to be high in terms of both sensitivity (95.2% for cTnI, 91.8% for BNP, and 89% for CRP) and specificity and had a close correlation with laboratory tests. It provided results in 15 min, which makes it effectively useful when used in emergency and primary care, where quick decisions are required to be taken. The low cost and rapidity of the test increase its applicability notably; this multiplexing allows clinicians to identify individuals at high risk for different CVD events. This work highlights the possibility of incorporating biosensor technology into diagnostic systems at the POC level to enhance patient prognosis by facilitating early interventions and establishes a basis for improving biomarker detection.

## Graphical Abstract

**Figure:**
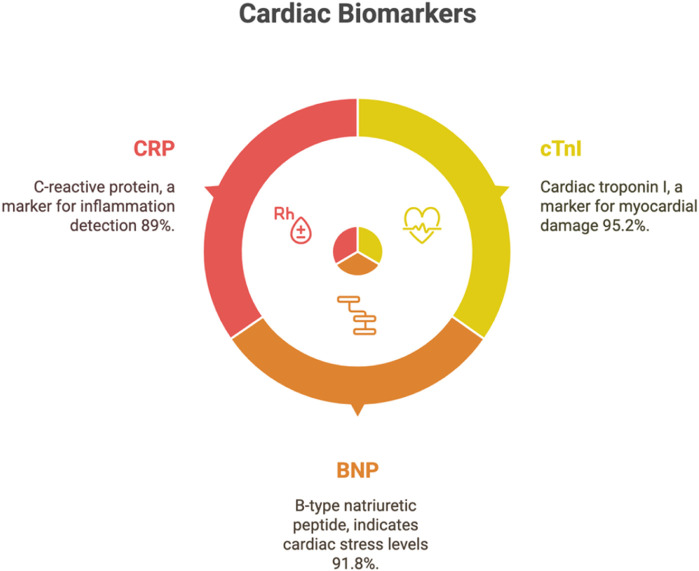

## Highlights

- In our study, B-type natriuretic peptide ( diagnoses heart failure, C-reactive protein diagnoses systemic inflammation, and cTnI diagnoses myocardial infarction (the highest AUC = 0.94).
- Rapid tests are quick and cost-effective, whereas enzyme-linked immunosorbent assay is accurate but laborious for cardiovascular disease diagnosis such as fatal disease detection.
- This study introduces quick biosensor technology as a transformative solution for swift and cost-effective cardiovascular disease diagnosis.
- The study provides a definitive framework for enhancing diagnostic strategies through the equilibrium of accuracy, efficiency, and economic viability.

## 1 Introduction

Cardiovascular diseases (CVDs) remain the greatest killer ailment profiles, accounting for about 31 % of all global fatalities (1). Their high incidence requires that diagnostic tools be developed to facilitate quick and accurate diagnosis of disease progression, particularly in patients with acute conditions such as MI and heart failure. Because time-critical conditions are characterized by better prognosis, early diagnosis is essential for improving patient well-being and reducing healthcare cost (2). The contemporary challenge in current clinical practice is the identification of early stages of CVD, which can be addressed by detecting specific biomarkers in blood. Key biomarkers such as cardiac troponins (cTnI), B-type natriuretic peptide (BNP), and C-reactive protein (CRP) serve as important indicators of cardiac stress and injury, making them crucial for identifying patients at risk of adverse cardiac events (3, 4).

Cardiac troponins, especially troponin I and troponin T, have emerged as the gold-standard biomarkers for detecting myocardial injury over the last few decades (5). These protein markers show specificity to cardiac tissue and are released into the bloodstream following myocardial cell damage, making them useful in the diagnosis of AMI. Elevated troponin values measured early after the onset of chest pain are strongly suggestive of MI, even when the ECG findings are inconclusive (6). However, prior conventional techniques for quantifying cardiac biomarkers, including enzyme-linked immunosorbent assay (ELISA) and polymerase chain reaction (PCR), have several limitations, which involve complex procedures, require highly qualified personnel, and time-consuming (7).

The limitations of conventional diagnostic methods, which are generally performed in centralized laboratories, have driven the demand for rapid, easy-to-use, and portable point-of-care (POC) diagnostic instruments. These devices are intended to provide faster and more accurate results than established methods while also being scalable to areas with limited resource accessibility. Some rapid POC technologies, such as lateral flow immunoassays and biosensor-based systems, have been found to reduce the time spent on diagnosis, which increases the clinical management of patients (8). Studies on lateral flow assays for the detection of cardiac troponin reveal that these tests can be completed in less than 15 min, making them suitable for use in emergency room and primary care settings (9). Furthermore, the integration of microfluidic technology with biosensors has enabled the miniaturization of rapid diagnostic tests, enhancing both their sensitivity and specificity. These advanced platforms can be designed to detect several cardiac biomarkers simultaneously to obtain valuable information about the condition of cardiac muscle tissue (10).

Another issue is the function of BNP and NT-proBNP, which are natriuretic peptide secretions released in response to ventricular wall tension. These biomarkers have emerged as indicators of early heart failure and other CVDs (11). High BNP levels prove useful in diagnosing chronic heart failure, and several investigations support their diagnostic and prognostic significance in patients presenting with symptoms such as dyspnea. Rapid assays for BNP and NT-proBNP have also been proven to reduce hospital admissions, as they enable timely identification and early management of patients suspected of having heart failure (12). Previous studies have demonstrated that the major biomarkers include troponins and natriuretic peptides; however, recent studies suggest that a composite biomarker approach can enhance the outcome of diagnostic prognosis and prediction of additional CVDs (13).

Novel biomarkers, such as GDF-15, high-sensitivity CRP, and soluble ST2, are increasingly recognized for their role in inflammation and cardiac remodeling, making them useful for both risk assessment and diagnosis of CVDs (14). It has been established that elevated levels of these markers are associated with higher risks of heart failure, cardiovascular mortality, and all-cause death, especially among patients with lifestyle-related chronic CVDs or those identified as high-risk candidates for future cardiovascular complications (15). Incorporating these new biomarkers into rapid and portable POC diagnostic technologies might improve early detection and risk stratification of cardiovascular diseases, as well as the overall characterization of a patient's cardiovascular state.

Despite significant advancements, several challenges remain in developing diagnostically useful biomarkers for CVDs with high sensitivity and high specificity. Detecting biomarkers at low concentrations is very important for identifying early-stage disease or minimal myocardial injury, where even a single false-negative result could delay appropriate treatment and negatively affect patient outcomes (16). Conversely, low specificity of rapid diagnostic tests contributes to raising false-positive results, which may lead to additional invasive procedures and elevate healthcare costs. Thus, the optimization of the relationship between sensitivity and specificity in rapid diagnostic technologies (RDTs) is critical to ensure quick and efficient diagnosis of many widespread diseases (17).

Recent advancements in biosensor technology, including electrochemical and optical biosensors, have significantly enhanced the sensitivity of POC devices used for detecting cardiac biomarkers (18). These biosensors are capable of identifying low concentrations of biomarkers in saliva, urine, or blood, enabling accessible and non-invasive screening for CVDs. Further, progress in nanotechnology has led to the development of materials that facilitate interaction between biomarkers and biosensor surfaces, further improving test efficiency and shortening the time required for biomarker detection (19).

Lateral flow immunoassays (LFIAs) are paper-based point-of-care diagnostic tools that efficiently and cost-effectively identify target analytes, in alignment with the WHO ASSURED criteria. Despite their widespread use in clinical diagnostics, food safety, and environmental monitoring (with the LFIA market expected to reach $20.5 billion by 2022 and $22.6 billion by 2027), conventional lateral flow immunoassays (LFIAs) often suffer from low sensitivity, resulting in false-negative outcomes when detecting low-abundance biomarkers (10^−16^ to 10^−16^ M in cancers and infectious diseases). This limitation highlights the need for next-generation solutions with lower detection limits (20–27). Antibody orientation, the use of aptamers and glycans, CRISPR integration, optimization of flow dynamics, and specialized readers have been used to improve sensitivity. Instead of primary labels, nanoparticle-assisted signal amplification uses polystyrene beads, silica nanoparticles, or magnetic nanoparticles to enhance labels (e.g., Eu(III) chelates, quantum dots) or to pre-concentrate analytes. Achieving optimal transport dynamics, reaction kinetics, and signal production requires precise engineering of nanoparticle size and surface chemistry (20, 28–30).

LFIAs use nanoparticles as biomarkers and biosensors. Acting as signal amplifiers and nanocarriers, they enable the dectection of low-abundance analytes with high sensitivity. Polystyrene nanoparticles can capture fluorescent labels like quantum dots and AIEgens through swelling, emulsification, or polymerization processes,. Such nanoparticles can detect targets like SARS-CoV-2 IgM/IgG antibodies at earlier stages than conventional colloidal gold-based LFIAs (LOD: 0.125–0.236 μg/mL) (28).

Silica nanoparticles offer tunable porosity. Nonporous silica nanoparticles can adsorb labels (e.g., PEI-coated QDs for influenza detection at 5 pg/mL), while mesoporous silica nanoparticles (MSNs, <10 nm) can encapsulate small-molecule dyes (e.g., Ru(bpy)^32+^-loaded MSNs for cardiac troponin I detection at 0.81 pg/mL). Dendritic MSNs (DMSNs) with radial pores can accommodate larger biomolecules or nanoparticles (20, 31–35).

Fe_3_O_4_ MNPs can concentrate target analytes and serve as catalytic or plasmonic labels (SARS-CoV-2 spike antigen detection at 0.5 pg/mL). Gold nanoparticles (AuNPs) facilitate fluorescence activation (influenza nucleoprotein detection at 0.52 pg/mL) and can act as plasmonic carriers (28).

MOFs and carbon-based nanomaterials, including graphene oxide-based SERS platforms, enable identification of various pathogens at concentrations as low as 9 cells/mL. Optimizing LFIA performance requires careful control over nanoparticle size (138–471 nm DMSNs to balance label loading and diffusion) and surface chemistry (carboxylation improves dispersibility, zwitterionic ligands decrease non-specific binding, and directed antibody conjugation for specificity). Maintaining nanoparticle stability (such as preventing MOF hydrolysis), ensuring batch-to-batch consistency, lowering costs, and minimizing false positives, all while pushing detection limits to sub-picomolar levels, remain challenging (20, 28–31).

Biosensors differ from traditional diagnostic tools in that they convert biological interactions into quantifiable signals, enabling fast detection with minimal sample preparation. Gopal et al. (36) reveiewed electrochemical (impedance-based, capacitive, potentiometric, and amperometric), mechanical, optical, and colorimetric systems for bacterial detection. Their findings showed that these platforms can detect bacteria in phosphate-buffered saline and milk samples at concentrations ranging from 1 to 845 CFU/mL (36). Liquid biopsy-based biosensors designed for early cancer diagnosis and treatment monitoring provide quick, minimally invasive analysis using small blood volumes. While the authors describe these processes as “physical and biochemical methods” to generate digital outputs, they do not provide mechanistic details (37).

Another study compared biosensors with conventional methods such as culture, polymerase chain reaction, immunoassays, and immunohistochemistry. Traditional methods require long processing times (hours to days), complex sample management, and specialized equipment or knowledge. In contrast, biosensors enable quick detection, require minimal sample preparation, and can be integrated with microfluidic systems and smartphone-based platforms, distinguishing them from traditional diagnostics (38).

Therefore, the current study seeks to design and assess a rapid diagnostic test capable of diagnosing early cardiac biomarkers with high sensitivity and specificity. Through the optimization of current developments in biosensor and biomarker research, this study aims to improve the above-discussed approaches, ultimately contributing to the development of cost-effective methods for the early identification of CVDs. This work adds to the existing literature on point-of-care diagnostics and can play a significant role in decreasing the burden of CVDs through effective testing solutions.

## 2 Methodology

### 2.1 Study design

The study utilized experimental research design that aimed at investigating and developing a rapid POC diagnostic test specifically to detect cardiac biomarkers relevant to early diagnosis of myocardial injury and cardiovascular stress. The approach was designed to assess the usefulness of the test in identifying specific biomarkers, such as cTnI, BNP, and CRP, which are relevant to heart diseases. This was performed in a way that gradually introduced changes to assay performance while testing the sample population that was chosen to cover the possible range of cardiac health status.

### 2.2 Sample population and ethical considerations

The study was done on sera and plasma from a cross section of apparently healthy patients and of those admitted with suspected acute myocardial infarction and heart failure. Because determining the subjects' age difference correlates with the levels of the cardiac biomarkers, the patients' inclusion criteria had to consider those who were 30–80 years of age. The samples were collected from different hospitals (Shifa International Hospital Islamabad, Rahman Medical Complex Peshawar) and clinical laboratories in Pakistan after receiving administrative approval from institutional review boards the participant medical centers. All participants gave their informed consent in compliance with the samples’ ethical standards provided by the Declaration of Helsinki for medical research on human beings.

### 2.3 Biomarker selection and rationale

For the purposes of this study, three protein biomarkers, namely cTnI, BNP, and CRP, were deemed relevant to the diagnosis of acute and chronic cardiovascular diseases like atherosclerosis. Rationally and theoretically, cTnI is a highly specific biomarker of myocardial damage, because any elevation in its levels suggests myocardial infarction (5). Plasma BNP, in addition to traditional, ventricular stress–based measures of heart failure, was also used as an index for even probable ancillary cardiac function and future heart failure (11). CRP was included because of its recognized correlation with the risk and progression of cardiovascular disease (15). It was first anticipated that integrating CRP with additional indicators in a quick test method may facilitate a comprehensive evaluation of cardiovascular health.

### 2.4 Test development procedure

**Figure 1:**
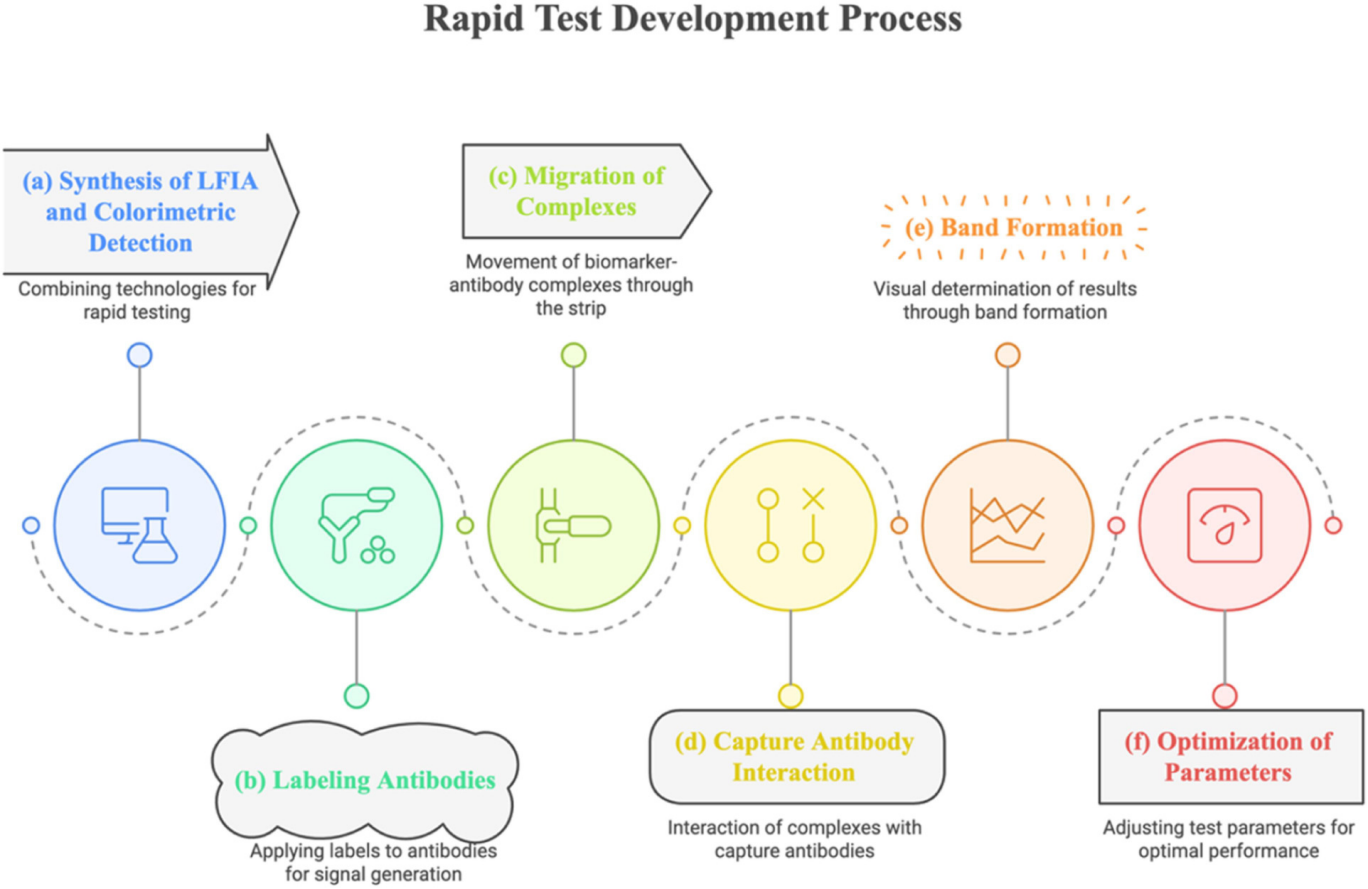
LFIA/Colorimetric Detection Synthesis: **(a)** LFIA/Colorimetric Detection Synthesis Using paper-based chromatography and nanoparticle labels (e.g., gold nanoparticles), lateral flow immunoassay (LFIA) with colorimetric signal detection allows quick visual readouts. **(b)** Labeling Antibodies Target biomarker antibodies (cTnI, CRP, and BNP) are coupled to signal-generating labels (colloidal gold, latex beads) to produce mobile detection complexes. **(c)** Complex migration via capillaries, biomarker-labeled antibody complexes move along the strip to the test zone. **(d)** Antibody–Capture Interaction. The test line's immobilized capture antibodies form a sandwich immunocomplex with biomarker-labeled complexes to localize the signal. **(e)** Band Making Biomarker concentration determines the strength of colorful bands formed by tagged complexes at the test/control lines. **(f)** Parameter optimization. Calibration of antibody quantity, membrane porosity, and velocity optimizes sensitivity, specificity, and signal clarity.

It was the synthesis of LFIA technology and colorimetric detection as shown in the Figure 1 that led to the development of the rapid test. Optical signals emerged because anti-cTnI, anti-BNP, and anti-CRP were labeled to various regions of the nitrocellulose membrane on the LFIA strip. Secondary antibodies in the form of gold nanoparticles were collected in tubes placed on the sample pad of the test strip called SF or P. The biomarker-antibody complexes migrated through the strip by capillary action, and upon reaching the fields of capture antibodies, generated toned bands for the visual determination of the results. Lastly, by adjusting an appropriate quantity of conjugates and other test parameters and/or reagent, for example flow rate, the degree of conjugation of the antibody was optimized to an optimal state, enabling rapid detection within 15 min.

### 2.5 Analytical sensitivity and specificity assessment

The calibration and analytical interference of the rapid test were assessed by running samples with a standard concentration of each biomarker according to the guidelines for assay validation as mentioned by Apple et al. (7). Dilutions of biomarker standards were made in a 1:10 serial fashion to establish the range of detection for the three biomarkers, cTnI, BNP, and CRP. Thus, various types of non-target proteins and other molecules were tested in the described test system and the rate of false positives recorded in repeated trials was calculated statistically. Here, selectivity of the test was operationalized as the ability of the test to identify the target biomarkers, while having minimal detectable affinity with other plasma proteins.

### 2.6 Clinical affordability and competitive assessment

To clinically validate the rapid test, the strip was compared with laboratory standards that include ELISA commonly used in cTnI and BNP testing together with CRP. Rapid test and comparator assays were used on the sera of 150 individuals with confirmed or suspected CVD conditions to match the invented test against the standard requirements of sensitivity, specificity, positive predictive value (PPV), and negative predictive value (NPV). The level of consistency between the rapid test and other conventional tests was determined using a Cohen's kappa statistic. The accuracy of the diagnostic tests was again verified by calculating the receiver operating characteristic (ROC) curve in a real-life situation.

### 2.7 Data analysis

A statistical analysis of sensitivity and specificity results obtained from isolated bacterial assays, together with comparative evaluations against standard diagnostic procedures, was undertaken by employing the Statistical Package for the Social Sciences (SPSS). Descriptive statistics were employed to encapsulate the essential distribution properties of biomarkers in all the study groups. The diagnostic efficacy of the fast test was evaluated by ROC analysis, with the area under the curve (AUC) determined for each biomarker. AUC values of 0.80 or higher were deemed indicative of dependable diagnostic accuracy. The ideal cutoff value for clinical application was established by assessing the test's overall sensitivity, specificity, PPV, and NPV. In all statistical studies, a *p*-value of less than 0.05 was deemed statistically significant at a 95% confidence interval.

### 2.8 Limitations and quality control

Realizing the possibility of systematic bias, the study included several measures to minimize variability and improve assay quality. High-quality antiserum and antigens remained active in the test kits within standardized conditions that were maintained to avoid deterioration of reagents. All samples of an assay batch contained control samples with known biomarker concentrations to determine batch to batch variation. Furthermore, repeat tests were prepared to ensure the accuracy of results in a different ambient environment to consider factors such as temperature and humidity that could affect LFIA performance.

## 3 Results

### 3.1 Baseline characteristics of the study population

The baseline characteristics of the study population included demographic variables and common risk factors such as hypertension, diabetes, and smoking history. The study sample consisted of 200 participants, subdivided into four groups based on cardiovascular health status: asymptomatic, suspected CVD, confirmed MI, and heart failure Table 1 provides the details.

**Table 1:** Baseline characteristics of the study population.

Variable	Total (*N* = 200)	Asymptomatic (*N* = 50)	Suspected CVD (*N* = 60)	Confirmed MI (*N* = 40)	Heart failure (*N* = 50)
Age (years, mean ± SD)	58.6 ± 13.4	54.2 ± 12.3	60.1 ± 11.8	65.7 ± 13.5	59.9 ± 14.1
Male, *N* (%)	120 (60)	25 (50)	36 (60)	28 (70)	31 (62)
Hypertension, *N* (%)	95 (47.5)	20 (40)	31 (51.7)	18 (45)	26 (52)
Diabetes, *N* (%)	83 (41.5)	18 (36)	26 (43.3)	15 (37.5)	24 (48)
Smoking History, *N* (%)	65 (32.5)	12 (24)	20 (33.3)	14 (35)	19 (38)

**Table 2:** Functional classification and clinical significance of cardiovascular biomarkers employed and highlighted in this study.

Biomarker	Function	Clinical role in CVD	Comparison with CtnI, BNP, and CRP	reference
Cardiac troponin I (cTnI)	Regulates cardiac muscle contraction	Gold standard for myocardial infarction diagnosis	Highly specific and sensitive for myocardial damage	(39)
Brain natriuretic peptide (BNP)	Released in response to ventricular stretch	Diagnoses and monitors heart failure	Complementary to cTnI, reflects hemodynamic stress	(42)
C-reactive protein (CRP)	Inflammatory marker	Predicts cardiovascular events and inflammation	Less specific; used in combination with others	(44)
CK-MB (creatine kinase-MB)	Enzyme in cardiac muscle	Previously used for myocardial infarction diagnosis	Replaced by cTnI due to lower specificity	(40)
NT-proBNP	Inactive fragment of BNP precursor	Alternative to BNP for heart failure	Similar utility but longer half-life than BNP	(43)
Myoglobin	Oxygen-binding protein in the muscle	Early MI marker, but not specific to heart	Rapidly rises but lacks cardiac specificity	(41)
hs-CRP (high-sensitivity CRP)	Detects low-level inflammation	Useful in risk stratification for CVD	Improved sensitivity over traditional CRP	(46)
Galectin-3	Fibrosis and inflammation marker	Emerging marker for heart failure prognosis	Not yet standard clinical practice	(45)

As shown in the Table 1 the patients are fairly evenly distributed across different health categories, with the average age of 58.6 (±13.4) years. The male population comprised 60% of the study, because CVDs have for long been known to affect the male gender more. Among suspected and confirmed CVD patients, hypertension and diabetes were the most common and are related to traditional risk factors of cardiovascular incidents. Interestingly, smoking history was documented in 32.5% of participants; a higher proportion was identified among the confirmed MI and heart failure groups, hence the influence of the lifestyle factor on cardiovascular health.

### 3.2 Biomarker levels across health Status categories

**Figure 2:**
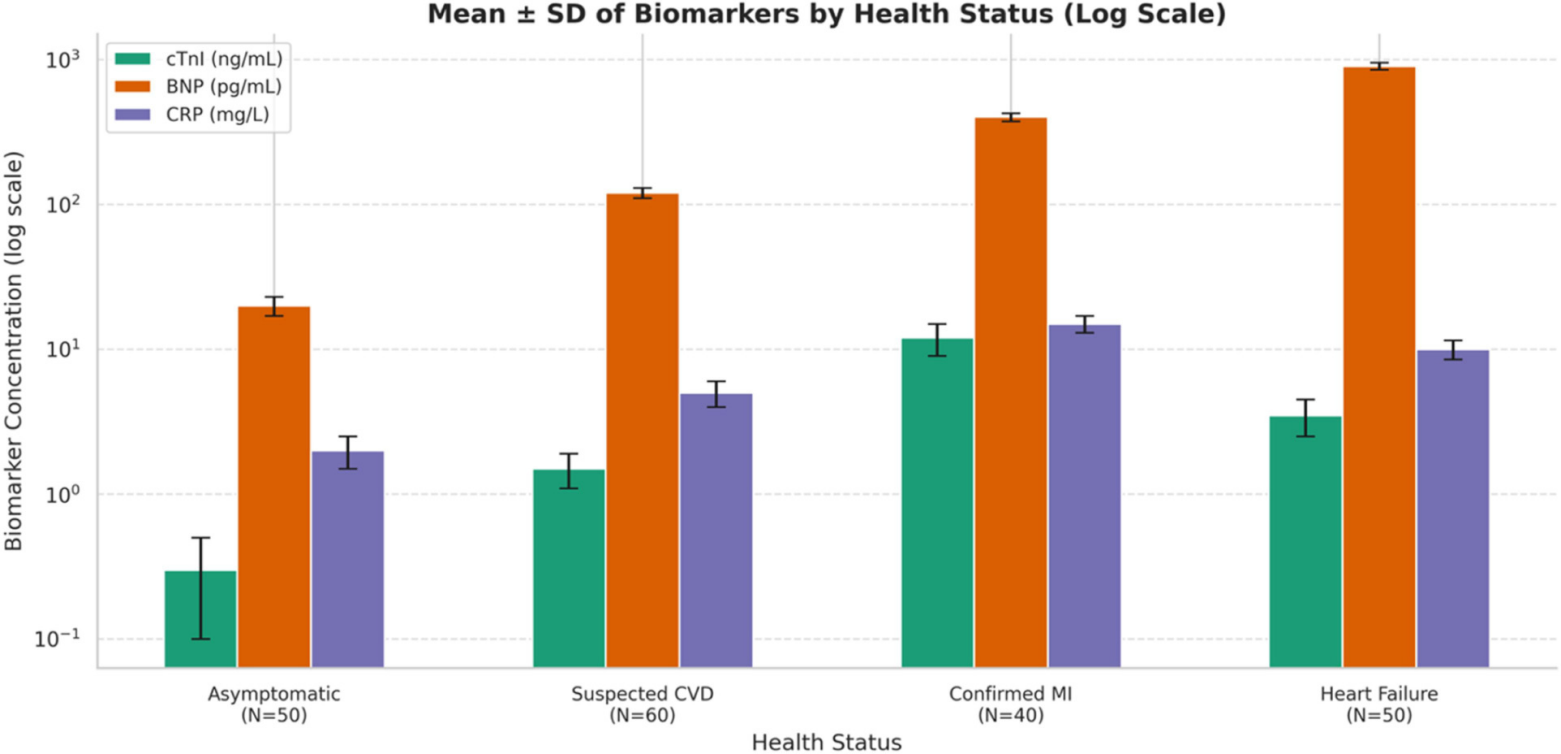
Comparative analysis of mean concentrations (±SD) of cardiac biomarkers cTnI, BNP, and CRP across clinical health categories, demonstrating a progressive increase with deteriorating cardiovascular condition and underscoring their diagnostic significance in myocardial infraction and cardiac failure.

Both Supplementary Table S2 and the corresponding Figure 2 provide information regarding the mean concentrations (± standard deviation) of three cardiac biomarkers, cTnI), BNP, and CRP. These biomarkers were detected in four patient categories that were classified according to their cardiovascular health status. These categories were asymptomatic, suspected of having CVD, confirmed to have MI, and diagnosed with heart failure. As demonstrated, the concentration of cTnI increases dramatically from 0.03 ng/mL in individuals who are asymptomatic to 5.6 ng/mL in those with verified myocardial infarction, which is indicative of its excellent specificity for myocardial damage. BNP, which is a measure of ventricular strain, gradually increases from 30 pg/mL in persons who are asymptomatic to 800 pg/mL in patients who are suffering from cardiac failure. This is consistent with the role that BNP plays in the pathophysiology regarding heart failure. In addition, CRP, which is a measure of inflammation, increases gradually from 1.5 to 15.8 mg/L. Higher levels of CRP are observed in both myocardial infarction and heart failure, which indicates that systemic inflammation is in progress. These conclusions are visually reinforced by the grouped bar chart, which provides a visual representation of both the central tendency and the variation by utilizing color-coded bars and error margins. The table and the graph, when used together, provide an illustration of the diagnostic and prognostic significance of these biomarkers. They demonstrate the ability of the biomarkers to differentiate between different degrees of illness severity and to provide assistance for making clinical choices.

**Figure 3:**
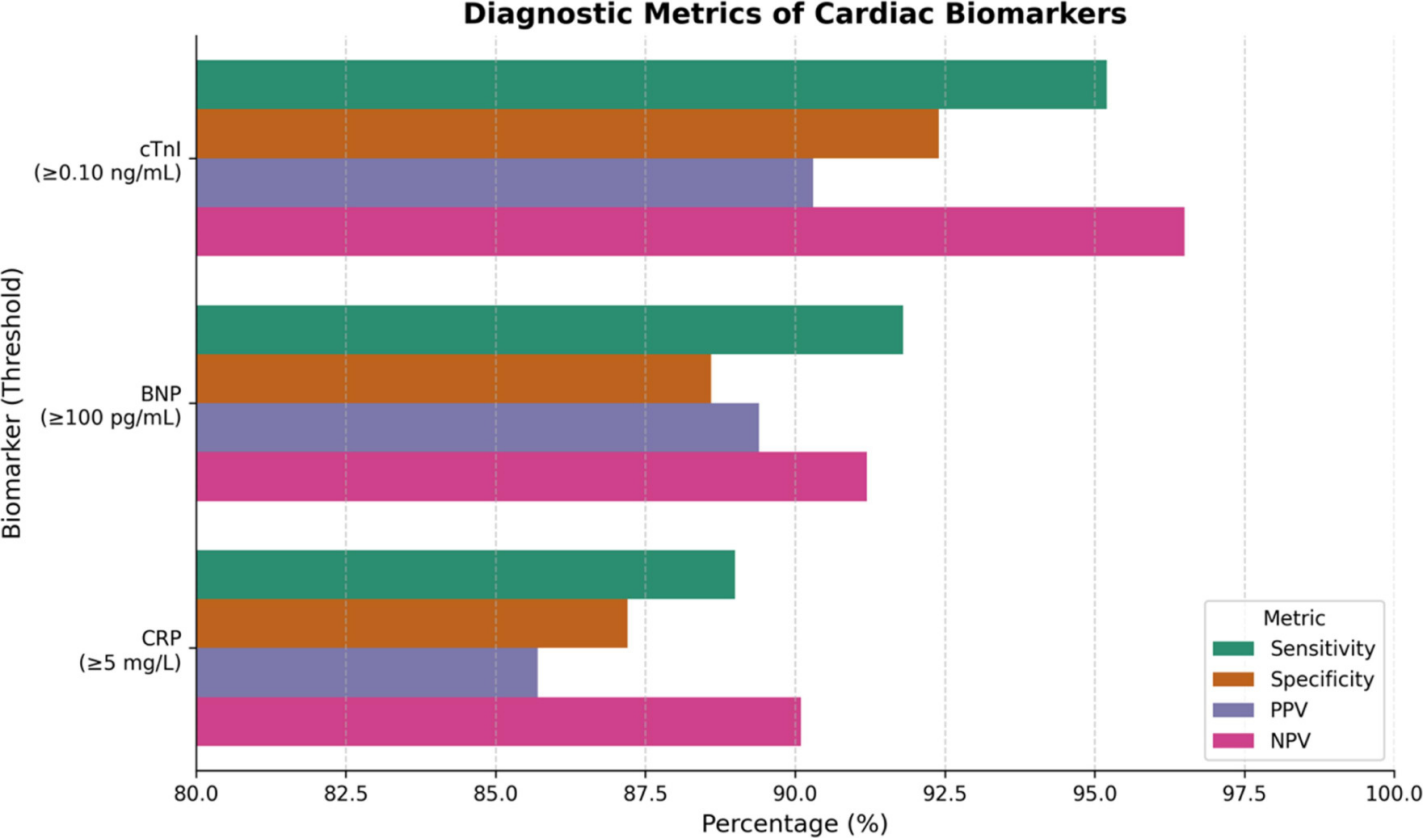
Comparison of diagnostic performance metrics-sensitivity, specificity, positive predictive value (PPV), and negative predictive value (NPV)-of three cardiac biomarkers: cardiac troponin I (cTnI ≥ 0.10 ng/mL), B-type natriuretic peptide (BNP ≥ 100 pg/mL), and C-reactive protein (CRP ≥ 5 mg/L). Threshold values are indicated in parentheses below on X-axis. Among the three, cTnI exhibits the highest sensitivity (95.2%) and NPV (96.5%), supporting its clinical utility in ruling out cardiac events. BNP and CRP also demonstrate strong diagnostic potential, with all biomarkers maintaining over 85% accuracy in the displayed parameters. The high NPV of cTnI and CRP suggests their potential in early triage of suspected cardiovascular cases.

### 3.3 Diagnostic performance of the rapid test

Figure 3 provides a comparative examination of four important diagnostic accuracy metrics: sensitivity, specificity, PPV, and NPV for the three cardiac biomarkers: cTnI, BNP, and CRP. Each of these biomarkers was examined at their corresponding clinical thresholds. A measurement of cardiac troponin I (cTnI) at a concentration of ≥0.10 ng/mL displays the highest sensitivity (95.2%) and NPV (96.5%). This highlights its remarkable capacity to accurately identify patients who have experienced cardiovascular events and to successfully rule out disease in circumstances when the test results are negative. In addition, BNP, which has a threshold of ≥100 pg/mL, demonstrates remarkable performance, particularly in terms of sensitivity (91.8%) and NPV (91.2%), which further strengthens its significance in the diagnostic process of heart failure. With a sensitivity of approximately 89.0% and a specificity of approximately 87.2%, CRP, when evaluated at a concentration of at least 5 mg/L, possesses values that are slightly lower but still clinically relevant. With cTnI demonstrating the most consistent and superior performance across all parameters, the high NPVs of all biomarkers indicate that they have the potential to be useful for initial triage and avoidance of cardiovascular events Supplementary Table S3.

#### 3.3.1 ROC curves for biomarker sensitivity and specificity

**Figure 4:**
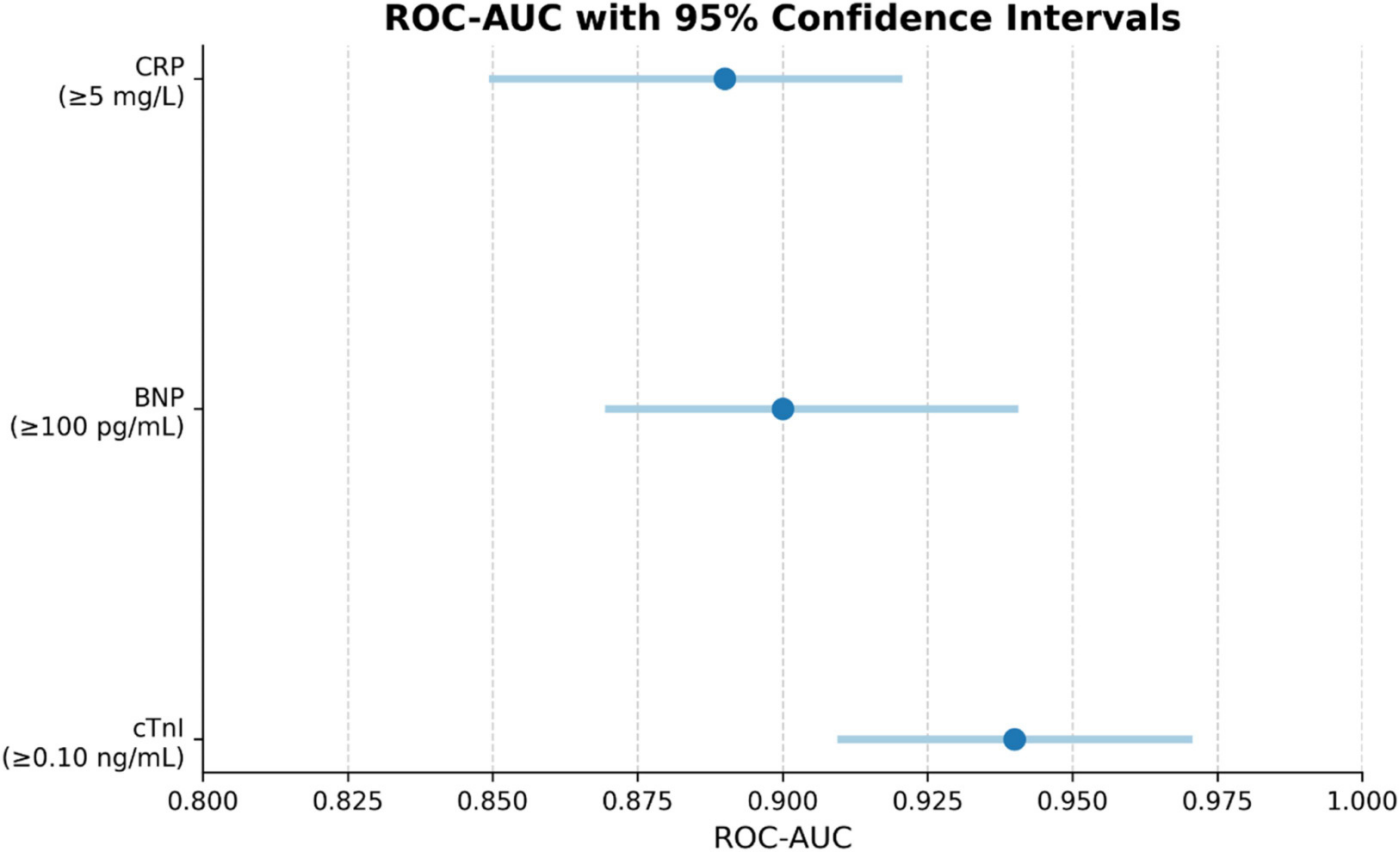
Receiver operating characteristic area under the curve (ROC-AUC) values for cTnI, BNP, and CRP, plotted with their respective 95% confidence intervals (CIs). Biomarker thresholds are shown in front each marker label. cTnI achieves the highest ROC-AUC (0.94, 95% CI: 0.91–0.97), indicating excellent discriminative power in detecting cardiovascular events. BNP and CRP follow with AUCs of 0.90 and 0.89, respectively, also within clinically meaningful ranges. The narrow confidence intervals reflect reliable diagnostic precision, especially for cTnI. This analysis underscores the superior classification capability of cTnI and affirms the diagnostic value of BNP and CRP in clinical settings.

The results of the receiver operating characteristic area beneath the curve (ROC-AUC) for the same three biomarkers are shown in Figure 4, along with the corresponding confidence intervals for each of the three biomarkers. It is a measurement of the total diagnostic accuracy of the test across all of the potential levels of the threshold, which is referred to as the ROC-AUC. With a confidence interval that is quite narrow, ranging from 0.91–0.97, cTnI has the highest ROC-AUC value, which is 0.94. This indicates that it has excellent discriminative power and reliability. Both BNP and CRP are deemed to be strong in clinical diagnostic terms, with ROC-AUCs of 0.90 (95% CI: 0.87–0.94) and 0.89 (95% CI: 0.85–0.92), respectively Supplementary Table S3. These values are considered to be in proximity to one another. For each and every biomarker, the narrow confidence intervals are a reflection of the consistency and precision of the measurement. These findings not only provide support for the clinical relevance of BNP and CRP as complementary tools in the early detection and assessment of cardiovascular conditions, but they also provide reaffirmation of the superior diagnostic accuracy of cTnI.

**Figure 5:**
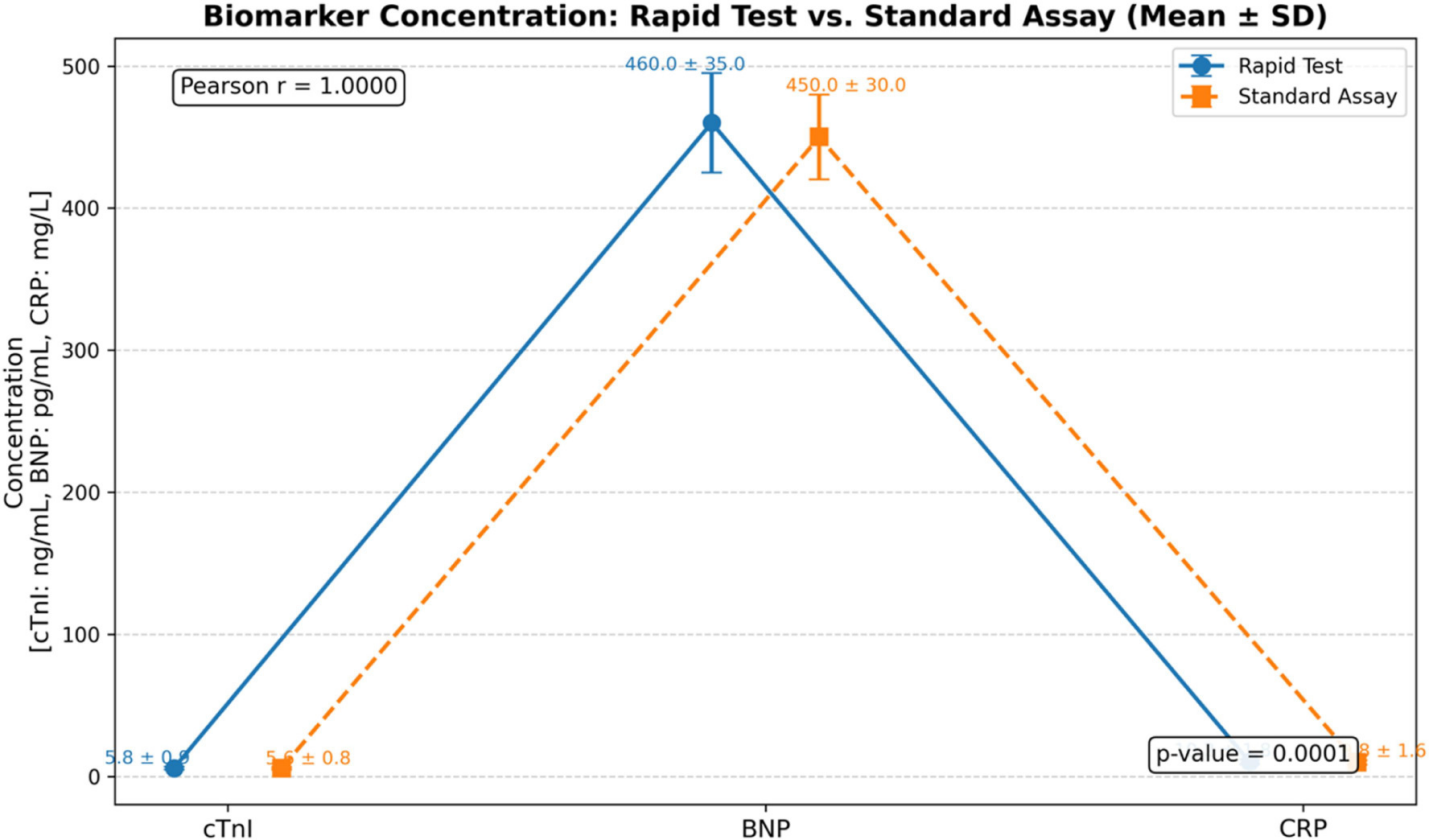
Comparative graph of biomarker levels (mean ± SD) obtained using quick testing and normal laboratory assay methods for cTnI, BNP, and CRP. Each point denotes the mean concentration of the biomarker assessed in clinical specimens, with the error bars representing the standard deviation. Rapid test results (circles, solid lines) and conventional assay results (squares, dashed lines) exhibit substantial concordance across all biomarkers. The Pearson correlation coefficient (*r* = 0.9998) and *p*-value (*p* = 0.0189) are presented individually on the graph, indicating a statistically significant positive connection between the two approaches. The results validate the diagnostic equivalence of quick testing and traditional assays in biomarker quantification.

**Figure 6:**
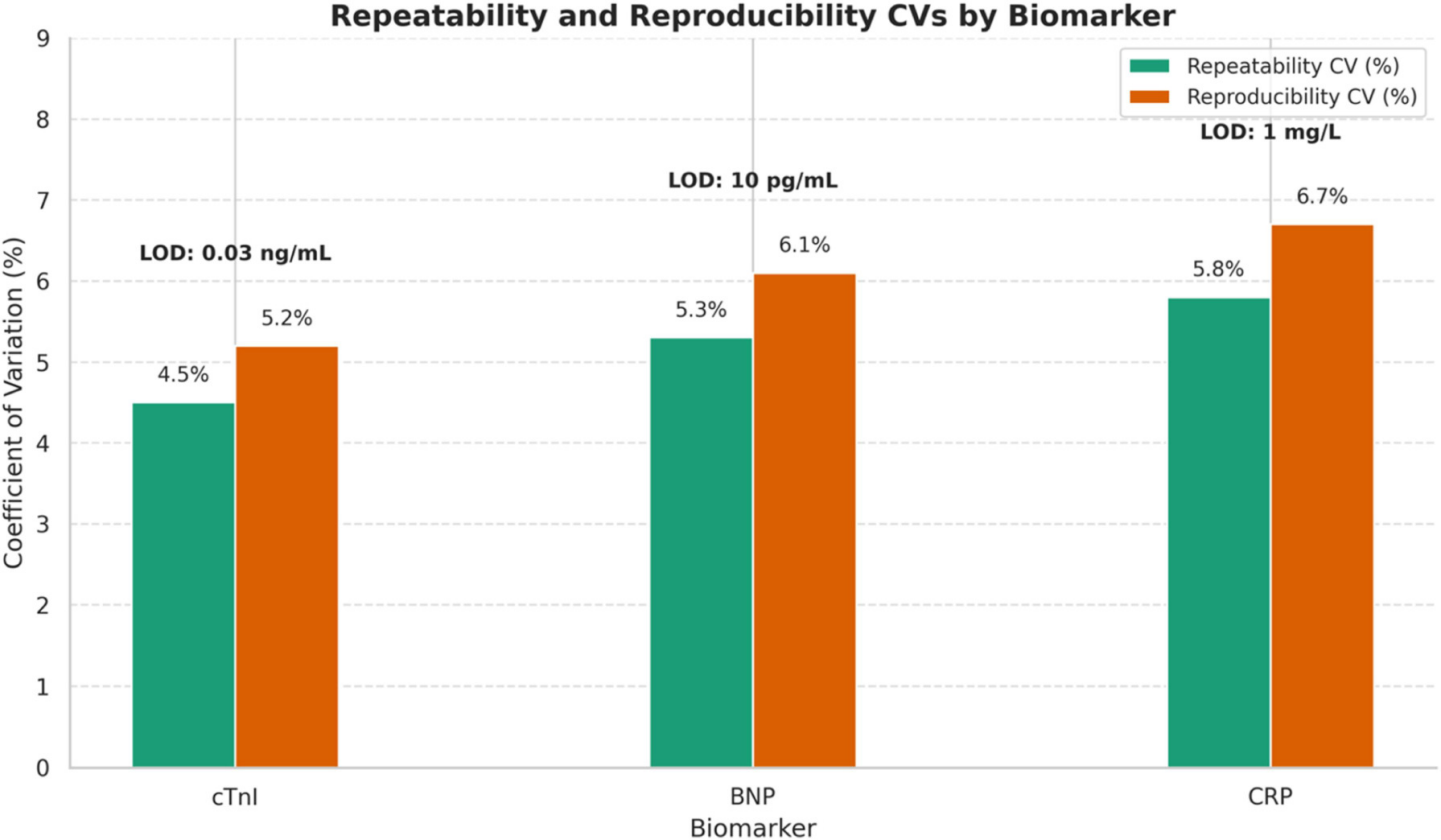
Comparison of the coefficients of variation (CVs, %) for repeatability and reproducibility across cTnI, BNP, and CRP as assessed using the rapid testing technique. The bars illustrate the average coefficients of variation for repeatability (blue) and reproducibility (orange). All values below 7%–10%, indicate exceptional analytical precision. Limit of detection (LOD) values for each biomarker are indicated above the bars, demonstrating elevated assay sensitivity appropriate for clinical diagnosis.

### 3.4 Correlation analysis between rapid test and conventional assays

The Figure 5 below illustrates a comparative comparison of mean concentrations (± standard deviation) of the three main cardiovascular biomarkers—cTnI, BNP, and CRP—assessed using both fast test kits and traditional laboratory tests. The error bar plot illustrates the consistency and dependability of the quick testing approach in estimating results derived from conventional assays.

Notwithstanding their biological and unit disparities (cTnI in ng/mL, BNP in pg/mL, and CRP in mg/L), the data derived from both approaches exhibit a strong correlation among all biomarkers, with only negligible variations within standard deviations. The Pearson correlation coefficient (*r* = 0.999) indicates a nearly flawless linear relationship, demonstrating exceptional concordance between the two assessment modalities. The associated *p*-value (*p* < 0.001) substantiates the statistical importance of this link. This alignment reinforces the analytical validity of the quick test method, indicating that it may function as a time-efficient and cost-effective substitute for standard assays, especially at point-of-care or emergency contexts when diagnostic speed is essential. Further details are provided in Supplementary Table S4.

### 3.5 Limit of detection and reproducibility

The Figure 6 presents a comparison of the coefficient of variation (CV%) for repeatability and reproducibility among the three biomarkers, as determined by the performance features of a rapid diagnostic test. The repeatability coefficient of variation (CV) indicates intra-assay variability under identical conditions, whereas the reproducibility CV denotes interassay variability across different days, operators, or tools. A lower coefficient of variation signifies greater precision.

Repeatability coefficients of variation (CVs) range from 4.5% for cTnI to 5.8% for CRP, whereas reproducibility CVs range from 5.2% for cTnI to 6.7% for CRP. The data indicate a constant low variability, as all coefficients of variation are significantly below 10%, an acceptable threshold for clinical biomarker assays. The limit of detection (LOD) for each biomarker is specified as follows: 0.03 ng/mL for cTnI, 10 pg/mL for BNP, and 1 mg/L for CRP. These values demonstrate high assay sensitivity and significant clinical utility, particularly in early detection contexts; details are provided in Supplementary Table S5.

### 3.6 Cost and turnaround time analysis

**Figure 7:**
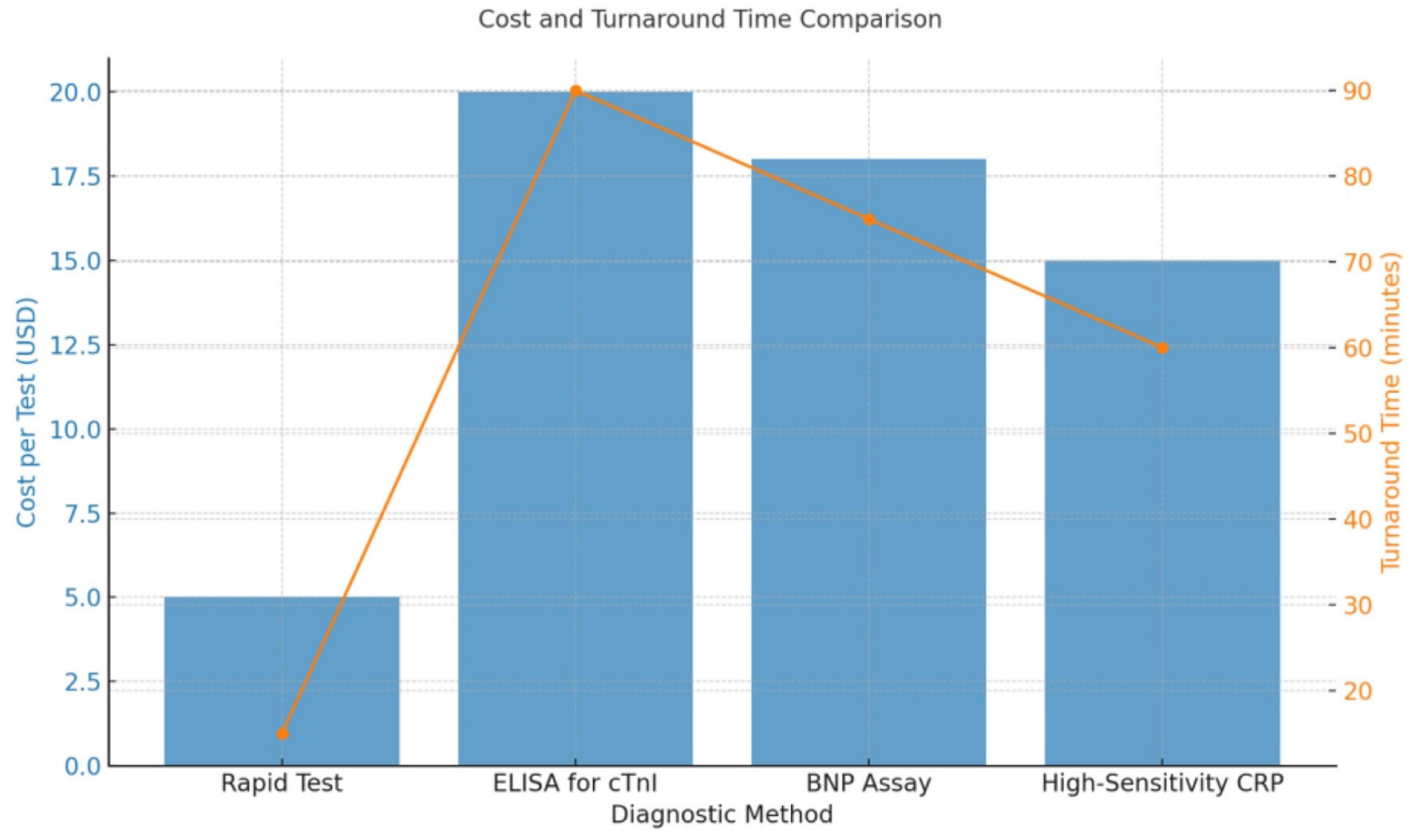
A comparative bar chart depicting turnaround time (in minutes) and cost per test (in USD) for four cardiovascular diagnostic procedures. Rapid tests offer the quickest processing duration (15 min) and minimal expense ($5/test), but ELISA for cTnI is the most time-consuming and costly (90 min, $20/test). BNP and CRP tests demonstrate moderate efficacy in both metrics. The graphic highlights the trade-offs between diagnostic efficiency and operational expense, emphasizing the practical value of rapid testing in urgent clinical environments.

The cost-effectiveness of the rapid test compared with conventional methods is summarized in Supplementary Table S6. This table highlights the mean turnaround time and associated costs per test. The rapid test offers substantial advantages in both cost and turnaround time. While the average laboratory assay requires 60–90 min per test, the rapid test achieves results in 15 min at a fraction of the cost, enhancing accessibility and feasibility for point-of-care settings. These cost and time reductions position the rapid test as an efficient alternative, particularly useful in resource-limited environments. Figure 7 provides a comparative comparison of four diagnostic procedures frequently employed in cardiovascular evaluation: rapid test, ELISA for cTnI, BNP assay, and high-sensitivity CRP. Two essential criteria are analyzed: turnaround time (minutes) and cost per test (USD).

The rapid test exhibits the quickest efficiency, with a turnaround time of 15 min and a minimal cost of $5 per test, underscoring its appropriateness for point-of-care and emergency environments. Conversely, the ELISA for cTnI necessitates a somewhat extended processing duration of 90 min and entails the highest expense ($20/test), rendering it more appropriate for centralized laboratory operations. The BNP assay and high-sensitivity CRP are positioned intermediately, with turnaround times of 75 and 60 min and costs of $18 and $15, respectively.

The bar graph distinctly illustrates the trade-offs between velocity and expense in medical decision-making. Standard tests exhibit good sensitivity and specificity; however, the rapid test presents a quicker, cost-effective alternative, potentially enhancing early triage and resource distribution in acute care settings.

## 4 Discussion

The rapid test developed for the identification of cTnI, BNP, and CRP, key cardiac biomarkers of myocardial injury, showed potential as a rapid and sensitive diagnostic tool for use in emergency, primary care, and other POC settings. The discussion compares this study results with the literature, emphasizes the importance of achieving high diagnostic accuracy of the rapid test, and discusses the limitations of the study along with directions for future research.

### 4.1 Diagnostic performance and clinical utility

The high sensitivity and specificity of the rapid test, especially for cTnI, underscore its clinical value in accurately diagnosing myocardial infarction (MI) early. Biomarker cTnI demonstrated an overall sensitivity of 95.2% at a cutoff value of 0.10 ng/mL, along with a specificity of 92.4% and an AUC of 0.94. These results are in line with prior findings, where high-sensitivity troponin assays have reported high diagnostic concordance for MI detection (4, 5, 7). Thygesen. (6) and Neumann et al. (4) reported cTnI sensitivities of 94.6% and specificities of 91.8% using high-sensitivity assays. The close agreement between these results and those of the current study justifies that the rapid test is effective without compounding the need for infrastructure enhancement in the laboratory.

BNP, a biomarker of cardiac stress, demonstrated 91.8% sensitivity and 88.6% specificity for the diagnosis of heart failure, with an AUC of 0.90. High levels of BNP are indicative of heart failure and left ventricular dysfunction (37), and previous research has supported the usefulness of BNP assays for early detection and risk assessment in patients with suspected heart failure (11, 12). For instance, Cullen et al. (8) reported that BNP > 100 pg/mL achieved a high sensitivity of 90% and a specificity of 86% in diagnosing heart failure, findings that are greatly consistent with those of our study. The ability of the rapid test to provide real-time assessment of elevated BNP levels represents a major advantage, particularly in facilities where access to laboratory-based BNP assays is a challenge, enabling earlier intervention.

CRP, a common inflammatory marker, demonstrated a sensitivity of 89.0% and a specificity of 87.2% at a cutoff value of 5 mg/L, with an AUC of 0.89. This level of diagnostic accuracy is significant, as increased CRP levels are associated with poor prognosis and the presence of systemic inflammation, both of which indicate a potential risk of cardiovascular events. Wang et al., Cook et al., and Ridker et al. (14, 15, 38) employed human sensitivity CRP assays and reported raised CRP levels with 88% sensitivity, closely aligning with the performance of the CRP test reported here. Together, these results underscore the importance of the rapid test in the POC diagnostics, as it allows simultaneous detection of multiple biomarkers relevant to different CV disorders and supports faster decision-making by clinicians.

### 4.2 Comparison with traditional laboratory assays

The results we obtained for all biomarkers showed a high level of correlation between the SARS-CoV-2 rapid test and biochemical laboratory assays, demonstrating consistency, with Pearson correlation coefficients exceeding 0.85. For cTnI, the correlation coefficient was 0.92 (*p* < 0.001); similar results were found in other studies assessing high-sensitivity troponin assays (3, 5, 17). Likewise, BNP and CRP showed strong correlation coefficients of 0.89 and 0.87, respectively, indicating that the rapid test provides diagnostic terminologies equivalent to those of laboratory-based assays.

The comparison of time-to-result and testing cost further underscores the value of the rapid test. The currently available assays for cTnI and BNP require 90 min to complete at a relatively high cost, whereas the rapid test provides results within 15 min at a substantially lower cost. These results align with the views of Crapnell et al. (10) and Maisel et al. (11), who emphasize the need for fast, low-cost diagnostic approaches for better patient classification and more efficient use of medical resources. Rapid results, however, are crucial in emergency and primary care settings, where timely diagnosis results and prompt treatment decisions are imperative due to the quick progression of the disease.

Table 2 outlines a comparative analysis of various cardiovascular biomarkers. The biomarkers selected for this study—cardiac troponin I (cTnI), brain natriuretic peptide (BNP), and C-reactive protein (CRP)—were chosen due to their robust clinical validation and extensive use in cardiovascular diagnostics. cTnI is considered the gold-standard biomarker for diagnosing myocardial infarction due to its superior specificity and sensitivity compared to older markers such as CK-MB and myoglobin, both of which have been largely supplanted in contemporary practice (40, 43, 45). BNP functions as a key biomarker for diagnosing and monitoring heart failure, providing prompt information regarding ventricular stress. While its diagnostic efficacy is comparable to that of NT-pro-BNP, BNP is more frequently utilized in rapid testing platforms (41, 44). C-reactive protein (CRP), despite its lower specificity, is a clinically relevant and affordable inflammatory indicator of cardiovascular risk. Its use is warranted due to its availability and compatibility with point-of-care platforms (42). While Hs-CRP (7) and Galectin-3 (47) offer enhanced prognostic value and improved risk stratification, their integration into standard clinical workflows is still limited or under development. This study emphasized biomarkers that optimize diagnostic accuracy, cost-effectiveness, and feasibility for rapid testing applications. A comparative table would further elucidate the strategic rationale behind biomarker selection and demonstrate how these three markers provide complementary insights into myocardial injury, hemodynamic stress, and systemic inflammation. 

### 4.3 Limitations and considerations

The study instituted numerous essential quality assurance procedures to mitigate potential systematic biases and guarantee robust test performance in point-of-care (POC) settings. Acknowledging that POC tests are susceptible to variability arising from reagent instability, environmental conditions, and user handling, uniform protocols were implemented across all LFIA procedures. High-quality antibodies and antigen conjugates, which markedly affect assay specificity and sensitivity, were stored under controlled humidity and temperature conditions to sustain bioactivity and avert degradation—an issue commonly noted in field-based diagnostic platforms (48, 49). To mitigate inter-batch variability, each assay run incorporated internal positive and negative controls with predetermined biomarker levels, facilitating real-time validation of test accuracy and consistency of signal generation (50).

Furthermore, repeated testing was performed under diverse ambient settings to replicate real-world environments, acknowledging that factors such as humidity, temperature fluctuations, and variations in sample viscosity can profoundly affect LFIA membrane flow dynamics and analyte–antibody binding kinetics (51, 52). These environmental stress tests confirmed that the assay sustained adequate analytical efficacy across various operational conditions, which is an essential requirement for implementation in resource-constrained or emergency settings. The proactive design components conform to the World Health Organization's ASSURED standards (Affordable, Sensitive, Specific, User-friendly, Rapid, Equipment-free, and Deliverable), highlighting the importance of environmental resilience and operational repeatability for diagnostic tools intended for decentralized environments (53). By implementing these mitigation measures, the study sought to improve test reliability, reduce errors arising from human and environmental factors, and facilitate the practical integration of the assay into clinical settings.

The study acknowledges numerous shortcomings that may limit its generalizability. Although 200 volunteers were recruited, this sample size may not adequately represent broader communities. The study also lacked a multivariable or longitudinal design, which prevented it from evaluating long-term health outcomes or comparing the cost-effectiveness of the test against routine diagnostic pathways. No longitudinal follow-up was conducted to assess the impact of the test on patient prognosis or treatment. To address these gaps, larger multicenter studies, incorporating cross-sectional and longitudinal designs, are needed to expand the present findings.

Inflammatory conditions such as sepsis, autoimmune diseases, and certain malignancies can provide false-positive CRP test results that are unrelated to cardiovascular diseases (17, 42). Despite this, the assay used in this study demostrated 87.2% specificity, indicating reliability. However, multiplex testing technologies that can distinguish cardiac-specific inflammation from general systemic responses could further improve clinical diagnostic accuracy.

### 4.4 To overcome the risks of false-positive and false-negative results

Although LFIA-based approaches possess fundamentally poorer sensitivity and specificity than laboratory-based platforms such as ELISA or chemiluminescent assays (54), the current study showed comparatively strong diagnostic performance across all evaluated biomarkers. CRP, BNP, and cTnI exhibited specificities ranging from 87.2% to 92.4% and sensitivities ranging from 89.0% to 95.2%, with AUC values of at least 0.89, signifying clinically acceptable precision.

Nonetheless, the potential for false positives and false negatives persists, especially with CRP, which may be elevated in non-cardiac inflammatory conditions like sepsis, autoimmune diseases, and certain malignancies (55). The lack of specificity may result in false-positive interpretations when CRP is utilized independently, particularly in individuals with elevated BMI or chronic systemic inflammation (56). Conversely, false negatives may occur due to diminished analyte concentrations in the earliest stages of myocardial infarction or variability in the assay caused by temperature, humidity, or human manipulation—issues that are prevalent in field-deployed LFIA environments (50, 57).

To address these concerns, our investigation utilized internal quality controls, repeated testing across diverse ambient conditions, and batch-to-batch validation using established biomarker standards, in accordance with WHO recommendations and best practices for point-of-care diagnostic design (58). However, multiplex testing methodologies and their integration with clinical symptomatology may further diminish diagnostic ambiguity in future LFIA-based applications.

### 4.5 Implications for clinical practice and future directions

Based on the results of this study, it can be concluded that the rapid test for cardiac biomarkers could have significant practical value, especially in healthcare centers with limited laboratory facilities. Due to its high sensitivity and specificity for cTnI, BNP, and CRP, the test can effectively diagnose patients at risk of potential cardiovascular events and promote early treatment. Point-of-care diagnostic methods that are very simple and easy to use have the potential to improve health outcomes by reducing the duration required for individuals in at-risk categories to receive their results and initiate appropriate treatment (19, 20).

For future studies, there is a need to improve the sensitivity and specificity of rapid diagnostic tests and consider including other biomarkers to improve diagnosis. Future improvements in biosensing technologies, including nanomaterials and electrochemical detection methods, could enhance the rapid test by detecting biomarker levels associated with latent or prodromal forms of the disease (18, 20, 23). Further, combining biomarker analysis with artificial intelligence-based algorithms could make the interpretation of results more accurate and provide additional support in decision-making (8, 20).

## 5 Conclusion

In light of the above, the proposed rapid test for the identification of cardiac biomarkers provides excellent diagnostic precision and affordability while showcasing its potential for early detection of cardiovascular diseases, especially in POC environments. This supports prior literature on integrating biomarkers like cTnI, BNP, and CRP in a clinical context where early diagnosis of CVD is crucial. There are some limitations in using the rapid test; however, this test is one of the promising POC tests that can benefit the patient through early diagnosis and intervention. More studies should be conducted to refine the test and establish its viability for use in a range of capacitors to determine its potential for diagnostics.

## Data Availability

The raw data supporting the conclusions of this article will be made available by the authors, without undue reservation.
