# Supplementary material for: Rapid diagnosis of different cardiovascular disease events from early released cardiac biomarkers, cTnI, BNP, and CRP, by biosensor technology
Source: Front Cardiovasc Med. 2025 Dec 17;12:1600695. doi: 10.3389/fcvm.2025.1600695 (PMC12754803; doi:10.3389/fcvm.2025.1600695)
Supplement: Supplementary file 1 [file Table1.doc]

**Rapid Diagnosis of Different Cardiovascular Diseases Events from the Early Released Cardiac Biomarkers: CtnI, BNP, and CRP by Biosensor Technology**

Razi Ullah^1, 2^, Mubassir Khan^3^, Wang Guixue^*1, 2^

1. Key Laboratory for Biorheological Science and Technology of Ministry of Education, State and Local Joint Engineering Lab for Vascular Implants College of Bioengineering Chongqing University Chongqing 400030, P. R.

China. Email; srazi1819@gmail.com.

2. Jin Feng Laboratory Chongqing 401329, P. R. China.

3. Key Laboratory of Biorheological Science and Technology Ministry of Education College of Bioengineering Chongqing University Chongqing, 400044 P.R. China.

Email; mubassirkhnn00@gmail.com

*Corresponding authors: wanggx@cqu.edu.cnt (GW)

Table 2: Mean Biomarker Concentrations across Health Status Categories

| **Health Status** | **Sample Size (N)** | **cTnI (ng/mL, mean ± SD)** | **BNP (pg/mL, mean ± SD)** | **CRP (mg/L, mean ± SD)** |
| --- | --- | --- | --- | --- |
| **Asymptomatic** | 50 | 0.03 ± 0.01 | 30 ± 5 | 1.5 ± 0.3 |
| **Suspected CVD** | 60 | 0.20 ± 0.05 | 105 ± 15 | 6.2 ± 0.9 |
| **Confirmed MI** | 40 | 5.6 ± 0.8 | 450 ± 25 | 15.8 ± 2.1 |
| **Heart Failure** | 50 | 1.2 ± 0.4 | 800 ± 40 | 9.6 ± 1.5 |

Table 3: Diagnostic Performance of the Rapid Test by Biomarker

| Biomarker | Threshold Level | Sensitivity (%) | Specificity (%) | PPV (%) | NPV (%) | ROC-AUC (95% CI) |
| --- | --- | --- | --- | --- | --- | --- |
| cTnI | ≥0.10 ng/mL | 95.2 | 92.4 | 90.3 | 96.5 | 0.94 (0.91-0.97) |
| BNP | ≥100 pg/mL | 91.8 | 88.6 | 89.4 | 91.2 | 0.90 (0.87-0.94) |
| CRP | ≥5 mg/L | 89.0 | 87.2 | 85.7 | 90.1 | 0.89 (0.85-0.92) |

For comparing biomarker levels derived from the rapid test against conventional methods, the following comparisons were made. Pearson’s r, mean, and standard deviation values for each biomarker are as following, which are summarized in **Table 4** below.

Table 4: Correlation and Concordance between Rapid Test and Conventional Assays

| Biomarker | Rapid Test Mean (± SD) | Standard Assay Mean (± SD) | Correlation Coefficient (r) | p-value |
| --- | --- | --- | --- | --- |
| cTnI | 5.8 ± 0.9 | 5.6 ± 0.8 | 0.92 | <0.001 |
| BNP | 460 ± 35 | 450 ± 30 | 0.89 | <0.001 |
| CRP | 10.2 ± 1.8 | 9.8 ± 1.6 | 0.87 | <0.001 |

**Table 4** demonstrates strong positive correlations between the rapid test and conventional assays, with correlation coefficients exceeding 0.85 for all biomarkers. The highest concordance was observed for cTnI (r = 0.92), suggesting the rapid test closely mirrors standard laboratory measurements for this biomarker. This high level of agreement reinforces the rapid test's validity as a practical alternative to traditional testing methods, particularly in settings where quick results are essential.

**Table 5: L**imit of Detection and Coefficient of Variation (CV) for Biomarkers

| Biomarker | Limit of Detection (LOD) | Repeatability CV (%) | Reproducibility CV (%) |
| --- | --- | --- | --- |
| cTnI | 0.03 ng/mL | 4.5 | 5.2 |
| BNP | 10 pg/mL | 5.3 | 6.1 |
| CRP | 1 mg/L | 5.8 | 6.7 |

**Table 6:** The cost-effectiveness of the rapid test compared to conventional methods is summarized in **Table 6**. This table highlights the mean turnaround time and associated costs per test. **Table 6** illustrates that the rapid test offers substantial advantages in both cost and turnaround time. While the average laboratory assay requires 60–90 minutes per test, the rapid test achieves results in 15 minutes at a fraction of the cost, enhancing accessibility and feasibility for point-of-care settings. These cost and time reductions position the rapid test as an efficient alternative, particularly useful in resource-limited environments.

Table 6: Cost and Turnaround Time Comparison

| Diagnostic Method | Mean Turnaround Time (minutes) | Cost per Test (USD) |
| --- | --- | --- |
| Rapid Test | 15 | 5 |
| ELISA for cTnI | 90 | 20 |
| BNP Assay | 75 | 18 |
| High-Sensitivity CRP | 60 | 15 |
